# Can technology help achieve sustainable intensification? Evidence from milk recording on Irish dairy farms

**DOI:** 10.1016/j.landusepol.2019.104437

**Published:** 2020-03

**Authors:** Lorraine Balaine, Emma J. Dillon, Doris Läpple, John Lynch

**Affiliations:** a Department of Economics, J.E. Cairnes School of Business & Economics, National University of Ireland - Galway, 49 Upper Newcastle, Galway, H91 YK8V, Ireland; b Agricultural Economics and Farm Surveys Department, Rural Economy and Development Programme, Teagasc, Mellows Campus, Athenry, Co., Galway, H65 R718, Ireland; c Department of Physics, University of Oxford, Parks Road, Oxford OX1 3PJ, United Kingdom

**Keywords:** Sustainable intensification, Technology impact, Sustainability indicators, Propensity score matching, Milk recording, Irish dairy farms

## Abstract

This article explores the potential of a farm technology to simultaneously improve farm efficiency and provide wider environmental and social benefits. Identifying these ‘win-win-win’ strategies and encouraging their widespread adoption is critical to achieve sustainable intensification. Using a nationally representative sample of 296 Irish dairy farms from 2015, propensity score matching is applied to measure the impact of milk recording on a broad set of farm sustainability indicators. The findings reveal that the technology enhances economic sustainability by increasing dairy gross margin and milk yield per cow. Furthermore, social sustainability is improved through a reduction in milk bulk tank somatic cell count (an indicator of animal health and welfare status). Conversely, milk recording (as it is currently implemented) does not impact farm environmental sustainability, represented by greenhouse gas emission efficiency. While the study shows that milk recording is a ‘win-win’ strategy, ways of improving current levels of utilisation are discussed so that milk recording achieves its ‘win-win-win’ potential in the future.

## Highlights

• On-farm technology uptake is important to achieve sustainable intensification.
• Milk recording is a useful tool to improve farm sustainability.
• Milk recording enhances economic performance and herd health.
• As currently implemented, milk recording does not impact milk GHG efficiency.
• Its application for breeding purposes could be fostered to improve GHG efficiency.

## 1 Introduction

Sustainable intensification is seen as an important means of addressing major challenges faced by the global food system, such as food security, environmental degradation and animal health and welfare concerns. As such, fostering its development has become an essential part of the agenda for policy makers and agri-food stakeholders (Department of Agriculture Food and the Marine, 2015; Food and Agricultural Organization of the United Nations, 2013; Franks, 2014). That is, attention has shifted from solely maximising agricultural productivity to optimising production across a wider set of economic, environmental and social sustainability objectives (Pretty et al., 2010). In other words, the intention behind sustainable intensification is to increase food production while simultaneously enhancing all three sustainability pillars (i.e., economic, environmental and social). This is not an easy task as it relies on systemic change at all levels of the food supply chain (Firbank et al., 2018) and conflicts between economic, environmental and social sustainability may arise in the intensification process (Bos et al., 2013; Dawkins, 2017).

At the farm level, sustainable intensification translates into increasing agricultural yields while using the same or a lower amount of inputs so that adverse environmental effects of agricultural production are reduced (Franks, 2014). It also entails ensuring that food is produced within a wider ethical framework (Garnett et al., 2013). When applied to livestock production, sustainable intensification relies on diluting the environmental costs of animal maintenance (environmental pillar) through production efficiency gains (economic pillar) (Crosson et al., 2011; Guerci et al., 2013), with socially acceptable standards of animal welfare (social pillar) (Garnett et al., 2013; Lebacq et al., 2013). Farm-level solutions must be found to help farmers undertake this challenge and achieve sustainable intensification in comprehensive terms. This article explores the potential of an agricultural technology to concurrently increase farm efficiency and provide wider environmental and social benefits in the context of dairy farming.

Technology adoption has traditionally been considered as a key mechanism for increasing farm productivity (Ali et al., 2018; Läpple and Thorne, 2018; Manda et al., 2016), but a stronger emphasis on identifying ‘win-win’ strategies to simultaneously pursue several sustainability objectives has emerged more recently (Hocquette et al., 2014; Llonch et al., 2017). While it is reasonable to expect concurrent sustainability benefits from the adoption of certain agricultural technologies (Guerci et al., 2013; Huijps et al., 2010b; Lanigan et al., 2018), claims must be further verified through rigorous empirical assessments (Balafoutis et al., 2017; Tullo et al., 2019). To the best of our knowledge, the current empirical literature provides limited indication as to which technologies can help resolve the sustainable intensification challenge across all three sustainability dimensions and lead to a ‘win-win-win’ (Llonch et al., 2017) scenario.

In the present article, we address this issue and focus on the case study of milk recording on Irish dairy farms. The need to find ‘win-win-win’ solutions to achieve sustainable intensification is particularly salient for the Irish dairy sector. Following the European Union (EU) milk quota abolition in 2015, a process of dairy expansion and intensification was set in motion in Ireland (Eurostat, n.d., 2020a, b) and poses significant sustainability concerns (Buckley et al., 2019; Lanigan et al., 2018). Milk recording is an agricultural technology, which provides per-cow information to farmers on a regular basis to support herd monitoring and decision making (Läpple et al., 2017). The use of milk recording information could lead to improve herd productivity and health, and reduce the environmental impact of milk production (Läpple et al., 2017). However, this ‘win-win-win’ potential has not yet been empirically verified.

Two main challenges arise from the estimation of technology impact on farm sustainability. On the one hand, new technologies can only be evaluated if their productive, environmental and social performances can be reliably estimated (Fumagalli et al., 2012), thus emphasising the need for relevant metrics to measure sustainability outcomes (Bélanger et al., 2012). Additionally, the quantification of environmental and social sustainability is limited by data availability, and subjectivity and complexity in delineating these terms (Lebacq et al., 2013). This inevitably results in a greater representation of easily-defined and -recorded economic performance and thus an imbalance between sustainability dimensions in the literature (Lebacq et al., 2013). We overcome this problem by using the rich and original 2015 Teagasc National Farm Survey (NFS) dataset, which comprises a nationally representative sample of 296 Irish dairy farms. Based on this data, we apply an indicator approach to measure farm sustainability (Hennessy et al., 2013; Lynch et al., 2016), with dairy gross margin and milk yield per cow for economic sustainability, GHG emission efficiency for environmental sustainability and bulk tank somatic cell count (BTSCC) of the milk produced for social sustainability.

On the other hand, as farmers ultimately decide whether or not to adopt a particular technology, self-selection must be accounted for when estimating technology impact. Drawing on previous theoretical and empirical literature (Dehejia and Wahba, 2002; Fentie and Beyene, 2019; Imbens and Wooldridge, 2009; Rosenbaum and Rubin, 1983; Schilling et al., 2014), we apply propensity score matching (PSM) to estimate treatment effects and assess milk recording’s ‘win-win-win’ potential. As a robustness check, additional estimation methods (i.e., inverse-probability weighting (IPW), regression adjustment (RA) and inverse-probability-weighted regression adjustment (IPWRA)) are implemented. Moreover, as PSM is based on the strong assumption that selection occurs only on observed characteristics, Rosenbaum bounds are estimated to test the sensitivity of treatment-effects estimates to hidden bias (Becker and Caliendo, 2007; DiPrete and Gangl, 2004; Rosenbaum, 2002).

The present study has direct policy relevance by addressing the topical issue of sustainable intensification and adds value to the existing literature in at least two ways. Firstly, it provides important insights on the suitability of a technology to overcome sustainability challenges arising from on-going agricultural intensification and can directly inform farmers’ adoption decision. The article takes an original approach by simultaneously investigating economic, environmental and social farm outcomes to explore the technology’s ‘win-win-win’ potential. Secondly, the study also contributes to the literature on the development and application of sustainability indicators by extending their use for measuring the impact of new technologies. A set of indicators has already been created specifically for the Teagasc NFS dataset (Hennessy et al., 2013; Lynch et al., 2016) and is utilised in this study. Thus far, farm sustainability indicators have mostly been used to assess time trends in sustainability (Buckley et al., 2016; Dillon et al., 2016a) or to compare production systems (Buckley et al., 2015); consequently, our application is new. Subject to data availability, this approach can be replicated in other agricultural settings.

The remainder of the article is structured as follows: section 2 reviews relevant literature. Section 3 introduces background information on Irish dairy expansion and milk recording. Section 4 outlines the methodology, followed by a description of the sustainability indicators used in the study and the data in section 5. Section 6 presents and discusses the results, while the final section provides the conclusions and policy implications.

## 2 Relevant literature

Agricultural technologies encompass a wide array of innovative practices implemented on farms. Among others, they can refer to new seed varieties, fertilisers or irrigation procedures (Doss, 2006; Kassie et al., 2018; Mutenje et al., 2016), new information and communication techniques to precisely inform management decisions (Barnes et al., 2019; Eastwood et al., 2012; Hennessy et al., 2016), milk meters (Eastwood et al., 2012; Hostiou et al., 2017), and growth hormones (Barham et al., 2004; McBride et al., 2004). In the literature, technology adoption has been identified as a main driver of farm productivity and profitability, and thus of farm economic sustainability (Ali et al., 2018; Läpple and Thorne, 2018; Manda et al., 2016). In the Irish context, Läpple and Thorne (2018) showed that innovation, as measured through an index that combines technology adoption, acquisition of knowledge and continuous innovation (Läpple et al., 2015), enhances farm economic sustainability, represented by profitability, productivity of land and market orientation.

However, technologies allowing for productivity gains might only resolve part of the sustainable intensification challenge. Not all technologies are equally suited to achieve this goal as their adoption might not always lead to synergies but also to trade-offs across sustainability dimensions (Dawkins, 2017; Lanigan et al., 2018; Llonch et al., 2017). For instance, from the perspective of environmental synergies, Lanigan et al. (2018) identified several efficiency measures (e.g., inclusion of white clover in pastures, use of sexed semen, improved genetic merit) that simultaneously increase farm economic performance and mitigate greenhouse gas (GHG) emissions from Irish milk production. Therefore, the adoption of these technologies can result in enhanced farm economic and environmental sustainability.

Additionally, technology-driven productivity gains can only show GHG mitigation benefits if emissions associated with intensification, particularly from off-farm sources, are offset by higher levels of efficiency (Crosson et al., 2011). In other words, increased productivity can mitigate GHG emissions of agricultural production if excessively high levels of external input application (e.g., concentrate feed and fertiliser) are avoided (Basset-Mens et al., 2009; Crosson et al., 2011). This can be a concern in intensive pasture-based production systems such as the Irish one. In fact, since higher-yielding cows might have greater nutritional requirements, not always achievable from grazing alone (Charlton et al., 2011), increased productivity might also lead to enhanced reliance on external inputs (Foote et al., 2015). In the New Zealand context, Basset-Mens et al. (2009) found that high-input pasture-based systems emit more GHGs per kg of milk produced than low-input ones. Therefore, not all technology-driven productivity gains might enhance farm environmental sustainability. Similarly, Lanigan et al. (2018) proved that not all mitigation strategies can increase farm efficiency nor be profitable (e.g., low emission slurry spreading techniques), which questions their voluntary adoption by farmers and ability to simultaneously reach economic and environmental sustainability objectives at the farm level.

From an animal welfare perspective, mastitis provides an interesting example of sustainability synergies and trade-offs. Mastitis is a contagious production disease widely spread on dairy farms at global scale (Sharma et al., 2011) and in Ireland (Geary et al., 2012; More et al., 2012), with adverse effects on animal welfare (Medrano-Galarza et al., 2012). In the last thirty years, its incidence has risen due to genetic selection heavily focused on milk production traits (Algers et al., 2009; Oltenacu and Broom, 2010). These traits are genetically antagonistic towards mastitis resistance, which is now increasingly taken into account in breeding programmes (Algers et al., 2009; Oltenacu and Broom, 2010). Decreasing mastitis occurrence and improving herd health and welfare are a promising path towards more sustainable dairy systems (Dawkins, 2017; Llonch et al., 2017). Indeed, the disease leads to substantial milk yield losses, decreased raw milk quality and avoidable culling decisions (Geary et al., 2012; Huijps et al., 2010b; Sharma et al., 2011). Beyond negative economic implications, mastitis also causes reduced GHG emission efficiency of dairy production (Özkan Gülzari et al., 2018; Özkan et al., 2015).

In that regard, routine hygiene measures such as carrying out post-milking teat disinfection were proven to be efficient to combat mastitis incidence in the Dutch (Huijps et al., 2010b) and Irish (Dillon et al., 2018) contexts. Thus, these results indicate that the uptake of such technologies could lead to increased herd health and welfare status, and thus farm social sustainability (Lebacq et al., 2013). Huijps et al. (2010b, a) also showed that these measures can be cost-efficient, notably by avoiding costs associated with mastitis, and suggest an economic benefit from implementing routine hygiene measures. Because of the association between mastitis, economic performance and GHG efficiency (Geary et al., 2012; Huijps et al., 2010b; Özkan Gülzari et al., 2018; Özkan et al., 2015; Sharma et al., 2011), technologies reducing mastitis occurrence could have a subsequent ‘win-win-win’ effect across sustainability dimensions.

So far, the empirical literature focuses on identifying ‘win-win’ technologies, notably for joint improvements in economic performance and animal health (Huijps et al., 2010b), or concurrent enhancements of production efficiency and greenhouse gas (GHG) emission efficiency (Lanigan et al., 2018). ‘Win-win-win’ potential is often discussed in reviews (Llonch et al., 2017; Tullo et al., 2019), but there is a lack of empirical proof to verify whether these technologies can indeed help achieve sustainable intensification in comprehensive terms (i.e., economic, environmental and social). We fill this gap by assessing milk recording’s ‘win-win-win’ potential. The methodology implemented in this study can be replicated to evaluate the ‘win-win-win’ potential of other technologies.

## 3 Background

### 3.1 Irish dairy expansion

The Irish dairy sector offers an excellent framework to explore sustainability issues associated with agricultural intensification, given large scale expansion post-EU milk quota abolition. Between 2010^1^ and 2017, dairy cow numbers and milk production increased by 33% and 40%, respectively (Eurostat, n.d., 2020a, b). Ireland’s export-oriented dairy sector gains from a competitive advantage in international markets by relying on a low-cost pasture-based production system, with further scope for and expectations of continued growth (Donnellan et al., 2015; Lanigan et al., 2018).

However, significant sustainability concerns arise from on-going, rapid growth (Buckley et al., 2019; Lanigan et al., 2018). Predicted growth in Irish agricultural output, mainly driven by increased dairy cow numbers and fertiliser use, is anticipated to result in a 9% rise in agricultural GHG emissions by 2030 relative to 2005 levels, thereby challenging the achievement of EU emission reduction targets (Lanigan et al., 2018). Agriculture is the largest single contributor to Irish GHG emissions by sector, accounting for about one-third of national emissions and half of emissions from non-Emission Trading Scheme (ETS) sectors^2^ (Duffy et al., 2017). In the context of the EU Effort Sharing Decision, the country must decrease non-ETS emissions by 30% by 2030 relative to 2005 levels (Environmental Protection Agency, 2018).

Moreover, even though the Irish grass-based milk production system is generally associated with high standards of animal welfare, dairy expansion and intensification could lead to challenges in that regard. There are international precedents for these concerns, notably in relation to udder and foot health (Lean and Playford, 2008; Algers et al., 2009).

Hence, this article addresses sustainability challenges at a critical time for the Irish dairy sector and is representative of situations faced by other agricultural sectors. It draws attention to the need to find ‘win-win-win’ technologies to achieve sustainable intensification and thus examines whether milk recording can provide solutions to alleviate sustainability conflicts.

### 3.2 Milk recording

Milk recording is an agricultural technology, which supports herd monitoring and farmers’ decision making (Läpple et al., 2017). Through the use of milk meters, it measures milk volumes and samples the milk from individual cows during milking, with two implementation options: 1) a manual option, for which a milk recording agent visits the farm to milk record, or 2) an electronic ‘Do-It-Yourself’ option, for which the farmer handles the recording himself/herself (in this case, appropriate training and support is provided by a technician) (Irish Cattle Breeding Federation, n.d., 2020a). Milk samples are then collected by milk recording organisations to be analysed. Result reports are returned to farmers through an online service or on a paper version, with further support to interpret them. They include detailed per-cow information about milk yield, constituents and somatic cell count (SCC) levels. Historical data is also reported. If utilised, milk recording data allows for better-informed decisions in several areas of farm management (Läpple et al., 2017).

In terms of reproductive management, milk recording information can help farmers identify the most profitable animals (i.e., high yielding, producing high-quality milk and with strong genetic merit) for breeding dairy replacements and informing culling decisions. In this way, farmers can increase milk quality and herd production performance (Läpple et al., 2017). Moreover, improved productivity through milk recording might be beneficial from a GHG mitigation perspective (Crosson et al., 2011; Guerci et al., 2013). Since agriculture is under pressure to reduce its emissions in Ireland (Buckley et al., 2019), active participation in the national GHG mitigation effort through the adoption of mitigation strategies by farmers is likely to increasingly gain in importance. Some caution must be exercised in verifying milk recording’s GHG mitigation potential. Because efficiency gains might lead to enhanced reliance on external inputs (Foote et al., 2015), off-farm reallocation of environmental burdens must be accounted for by applying a cradle-to-farm gate GHG estimation approach (O’Brien et al., 2014b).

In terms of animal health management, milk recording allows for the monitoring of mastitis by providing SCC readings for individual cows. Mastitis is mainly caused by bacterial infection in the udder and leads to elevated SCC (Geary et al., 2012; Huijps et al., 2010b; Sharma et al., 2011). The threshold of 200,000 somatic cells per millilitre is generally accepted as an indicator of mastitis incidence (International Dairy Federation, 1997). Thus, SCC can be used to reliably detect mastitis incidence, even when clinical symptoms are not yet observable, and react accordingly (AHI, 2012; Sharma et al., 2011). Previous research has concluded that Irish dairy farmers tend to adopt a reactionary as opposed to a precautionary approach when managing mastitis, responding mainly to an indication of infection (Dillon et al., 2018). This suggests that (subclinical) mastitis is not identified or treated on time and underlines milk recording’s potential herd health benefits.

From an implementation perspective, milk recording does not require any upfront investment. It costs approximately €12 per cow to milk record six times per year (Irish Cattle Breeding Federation, n.d., 2020a), but farmers can do it as frequently as they wish. The technology is risk-free and easy to use because it necessitates little or no technical skills. Implementation does not generally disrupt the milking routine, although it does slightly lengthen the milking task and requires the presence of an extra person in the parlour.

Finally, the use of milk recording is less prevalent in Ireland compared to some EU counterparts, with the technology utilised on 52% of Irish dairy cows in 2015 as opposed to 86% and 69% in Germany and in France, respectively (ICAR, n.d., 2020). Reasons for these stark differences in adoption rates across countries are not fully understood, but one possible explanation may be that the benefits of milk recording are not clear or, alternatively, not effectively communicated in Ireland. This article contributes to resolving these issues by evaluating the technology’s impact across all sustainability dimensions. We define adopters as farmers who milk record at least once per year and non-adopters as farmers who do not milk record at all.

## 4 Methodology

### 4.1 The impact evaluation problem

Ideally, the impact of technology adoption would be estimated by calculating the difference Δ in outcome at time t between a state where the farmer adopts the technology ($Y_{t}^{1}$) and a state where he/she does not adopt the technology ($Y_{t}^{0}$), as follows:

(1) ATT=E(ΔT=1),

where ATT is the Average Treatment Effect for the Treated (i.e., the average return only for the pool of adopters) and T indicates whether the technology has been adopted (T=1) or not (T=0). However, calculating Δ is impossible as farmers can only be observed in one of the two states (i.e., adopter or non-adopter), thus highlighting the need to construct counterfactuals (Blackman and Naranjo, 2012; Imbens and Wooldridge, 2009).

This problem could be solved by randomly assigning treatment, such that $E\left(Y_{t}^{0},T=0\right)=E\left(Y_{t}^{0},T=1\right)$, and Eq. (1) would become:

(2) $$ATT=E(Y_{t}^{1}|T=1)-E(Y_{t}^{0}|T=0).$$

However, when using non-experimental data, individuals choose their treatment rather than being randomly assigned, which introduces well-known self-selection bias. In other words, technology adoption could lead to enhanced sustainability, but ‘better’ farmers are also more likely to adopt the new technology. This suggests the presence of initial differences between adopters and non-adopters, which may invalidate causal comparisons of outcomes by treatment status (Imbens and Wooldridge, 2009).

While several methods allow to control for self-selection bias and estimate treatment effects (Imbens and Wooldridge, 2009), data availability often limits choice. In absence of suitable panel data or credible instruments, PSM has emerged as a popular approach in agricultural contexts (Fentie and Beyene, 2019; Schilling et al., 2014).

### 4.2 Propensity score matching

In this article, we apply PSM and estimate the ATT of milk recording adoption on farm sustainability. Assuming that selection occurs only on observables, adopters and non-adopters with the same probability p of adopting the technology (T=1), given a set of covariates X, can be compared and matched (Rosenbaum and Rubin, 1983). Under this assumption, the within-matched-pair difference in outcomes is then attributable to the technology’s impact and treatment effects are estimated by averaging within-matched-pair differences in outcomes (Imbens and Wooldridge, 2009). Thus, Eq. (2) becomes:

(3) $$ATT_{PSM}=E(Y_{t}^{1}|T=1,p)-E(Y_{t}^{0}|T=0,p).$$

Additionally, we use PSM to predict for each farmer both potential outcomes ($Y_{t}^{0}$ and $Y_{t}^{1}$), adjusted for observables, and estimate potential outcome means (POM) for the whole population, such that ${POM}^{k}=E(Y_{t}^{k})$, where $k=\left\{0;1\right\}$. In this way, ATTs can be expressed as a percentage of potential outcome means.

The use of PSM involves a series of practical choices before estimating ATTs. First, we estimate propensity scores pi=P(T=1Xi) for each farmer i with a logit model (Dehejia and Wahba, 2002). $X_{i}$ are a set of farm and farmers’ characteristics that simultaneously affect milk recording adoption and farm sustainability, but are not impacted by adoption status (Caliendo and Kopeinig, 2008). This model is equivalent to an adoption decision model and is reported in Appendix A (Model 1). Moreover, the model specification must meet two requirements: the overlap assumption and the balancing property (Caliendo and Kopeinig, 2008). For the overlap assumption, a match with similar propensity score value must be found for each adopter. We ensure that, for each farmer i, $p_{i}$ is included between 0 and 1 and that there is significant overlap between adopters’ and non-adopters’ propensity scores by plotting their distribution by treatment status (Dehejia and Wahba, 2002; Schreinemachers et al., 2016). The plot is presented in Appendix B. For the balancing property, estimated propensity scores must balance the covariate distribution between adopters and non-adopters. We base the balance diagnosis on standardised differences. The aim is to reach post-matching values of at most 10% across covariates and on average over all covariates (Austin, 2009; Schreinemachers et al., 2016). Appendix C displays these standardised differences before and after PSM and shows significant reduction in covariate imbalance after PSM.^3^

Second, following the propensity score estimation, we perform Nearest-Neighbour Matching (NNM), which matches adopters to their closest non-adopter(s) in terms of propensity score value. This method is common in PSM (Dehejia and Wahba, 2002; Schreinemachers et al., 2016). Its implementation relies on two practical choices that entail a trade-off between precision of treatment effect estimate and bias reduction (Dehejia and Wahba, 2002). One must select the number of matches for each adopter and whether to match with or without replacement (i.e., whether to match non-adopters more than once). To reach the best trade-off, we choose two matches with replacement^4^.

Finally, we evaluate matching quality by ordering propensity scores from lowest to highest and plotting them for adopters and matched non-adopters (Dehejia and Wahba, 2002). The plot is displayed in Appendix D and indicates that matching was performed successfully.

### 4.3 Sensitivity analysis

As a robustness check, we estimate ATTs with alternative treatment-effects estimation methods, including IPW, RA and IPWRA (StataCorp, 2013). We then check whether resulting ATTs differ significantly by estimating the overall coefficient of variation (CV) (Schreinemachers et al., 2016). The latter measure shows the extent of variability relative to the mean and is calculated as the ratio of the standard deviation to the mean for the results of each outcome variable. This robustness check ensures that ATT results are insensitive to changes in the estimator (Schreinemachers et al., 2016).

Furthermore, we cannot directly assess whether bias was introduced by the presence of unobservables that (are likely to) affect the adoption decision (e.g., farmers’ ability, motivation). In fact, one of the main shortcomings of PSM is that selection is assumed to occur only on observables and that this hypothesis cannot be formally tested. In this context, Rosenbaum bounds are meant to simulate the effect of unobservables on treatment effect estimates and to test the sensitivity of PSM results to hidden bias (Rosenbaum, 2002). Following Becker and Caliendo (2007), the adoption probability for each farmer i is given by:

(4) pi=P(T=1Xi)=F(βXi+γui),

where $u_{i}$ is an unobserved variable and γ the effect of $u_{i}$ on the adoption decision. If the analysis is free of hidden bias, γ will be zero and the adoption decision will be determined only by $X_{i}$. Conversely, if unobservables affect the adoption decision, farmers i and j with the same observed X will have different probabilities of adopting milk recording. Assuming F is the logistic distribution, the odds ratio Γ between both farmers i and j is given by:

(5) Γ=pi(1-pi)pj(1-pj)=pi(1-pj)pj(1-pi)=exp(βXi+γui)exp(βXj+γuj)=exp(γ(ui-uj)).

if farmers i and j have identical observed X (as assumed in PSM). In other words, both farmers i and j differ in their odds of adopting milk recording by a factor equal to Γ. If there are either no differences in unobserved variables ${(u}_{i}=u_{j})$ or unobservables have no impact on the adoption probability (γ=0), then Γ is 1, implying that PSM is successful in estimating unbiased effects. Conversely, if an unobserved characteristic impacts the adoption probability (γ≠0), it causes the odds ratio of the adoption decision to differ between farmers i and j by a factor Γ different than 1 and ATTs are likely to be biased. More formally, the Rosenbaum bounds approach is based on a Wilcoxon signed-rank test. At each Γ value, hypothetical significance levels are calculated and represent the upper and lower bounds of the ATT significance level in case of endogenous adoption decision (DiPrete and Gangl, 2004). Critical Γ values at which the p-values exceed the 10% threshold correspond to the magnitude of hidden bias required to alter PSM results and question causal inferences.

## 5 Outcome variables and data description

### 5.1 Choice of sustainability indicators and data

Evaluating the impact of milk recording on farm sustainability is a complex undertaking as it relies on the quantification of farm sustainability outcomes. Measuring farm sustainability through indicators has become a popular approach (Dillon et al., 2016a), which we apply in this article through the Teagasc NFS data from 2015. Sustainability indicators are “quantifiable and measurable attributes of a system that are judged to be related to its sustainability” and can help reveal movements in “the desired or undesired direction” in the data (Dillon et al., 2016a: 32). In this manner, they can provide useful insights to guide public policy (Bélanger et al., 2012; Dillon et al., 2016a; Fumagalli et al., 2012), such as exploring the sustainability potential of new technologies before encouraging widespread adoption.

The Teagasc NFS data is a rich and original enhancement of the data recorded for EU Farm Accountancy Data Network purposes^5^ (Dillon et al., 2016a). Professional farm recorders collect the data annually through face-to-face surveys over two to three farm visits. Overall, a randomly selected sample of approximately 900 farms participates each year on a voluntary basis and is nationally representative of the Irish farming population. Respondents are classified into six farming systems depending on the main source of farming income: dairy, cattle rearing, cattle other, sheep, arable and mixed livestock. In this article, we focus on a sub-sample of 296 dairy farms from the 2015 survey, for which the data required to calculate farm sustainability indicators is recorded.

Within the set of farm sustainability indicators available through the Teagasc NFS, we choose the ones that are suitable in the context of milk recording (Bélanger et al., 2012). More specifically, selected indicators must be able to capture changes in farm sustainability related to the technology’s uptake, they must be linked with the overall objective of achieving sustainable intensification and they must also be relevant for potential users (e.g., farmers, extension agents, policy makers). Therefore, we focus on dairy gross margin and milk yield per cow to represent economic sustainability, GHG emission efficiency of milk production as an indicator of environmental sustainability and BTSCC of the milk produced to measure social sustainability.

Dairy gross margin per cow is calculated as gross output minus direct production costs of the dairy enterprise on a per-cow basis. Milk yield per cow is measured as the total amount of milk produced on the farm, including milk sold and milk fed to other livestock, divided per cow.

GHG emission efficiency of milk production is the GHG emissions per kg of unit produced and thus a measure of farm environmental sustainability. For each farm, estimates of agricultural GHG emissions are derived by using a cradle-to-farm gate life cycle assessment (LCA) approach developed by O’Brien et al. (2010, O’Brien et al., 2014a, b). The LCA methodology is internationally standardised (International Organization of Standardization, 2006a, b) and specific guidelines have been developed to assess GHG emissions of milk production (British Standards Institute, 2011; Carbon Trust, 2010; International Dairy Federation, 2015). Following these guidelines^6^, a holistic-systems approach to quantifying GHG emissions throughout the production process is adopted (i.e., from off-farm production and acquisition of inputs to on-farm production of milk). All off- and on-farm GHG emissions associated with dairy production are modelled by combining the information from the Teagasc NFS dataset and emission factors estimated using the Intergovernmental Panel on Climate Change (IPCC) guidelines or other resources in the literature (Dong et al., 2006; Duffy et al., 2017)^7^. Emissions are then converted to total kg of carbon dioxide equivalent (CO_2_e) using the 100-year Global Warming Potential (Forster et al., 2007), as used for national emissions reporting. They are reported per kg of Fat-Protein-Corrected-Milk (FPCM) (International Dairy Federation, 2015), which controls for differences in milk solids between individual farms.

BTSCC depicts risk for mastitis incidence at the herd level and thus general herd health status (Geary et al., 2012; More et al., 2012; Sharma et al., 2011). It reflects farm herd health management and animal welfare levels (Huijps et al., 2010b; Medrano-Galarza et al., 2012), which is an important component of the social sustainability of livestock-based agricultural systems (Lebacq et al., 2013). Through the Teagasc NFS, monthly data on herd-level SCC based on milk bulk tank readings is available and is utilised to calculate a yearly weighted average so that the seasonality in Irish milk production is accounted for (Dillon et al., 2015).

### 5.2 Descriptive statistics

We assess the difference in sustainability performance between milk recording adopters and non-adopters in Table 1 by dividing farms by adoption status and running a bivariate analysis. The results show that adopters have higher dairy gross margins and milk yields per cow, are more efficient in terms of GHG emissions and achieve lower BTSCCs.

**Table 1:** Sustainability performance of Irish dairy farms, by technology adoption status.

Outcome variable	Non-adopters (*n* = 151)	Adopters (*n* = 145)	All farmers (*n* = 296)	*t*-test
Dairy gross margin per cow (€ / cow)	1,004.62 (278.38)	1,132.78 (264.52)	1,067.40 (278.71)	−4.06***
Milk yield per cow (l / cow)	5,155.42 (956.45)	5,803.22 (894.36)	5,472.76 (980.22)	−6.01***
Agricultural GHG emissions per kg of output (kg of CO_2_e / kg of FPCM)	1.20 (0.24)	1.12 (0.19)	1.16 (0.22)	3.37***
BTSCC (‘000 cells / ml)	192.16 (72.60)	156.30 (56.37)	174.59 (67.48)	4.73***

Notes: Means and standard deviations in parentheses.

***, **, and * significant at the 1%, 5%, and 10% level, respectively.

The summary statistics reported in Table 2 reveal that adopters and non-adopters also differ in terms of farm and farmers’ characteristics, which suggests that self-selection may be at play. Adopters have larger and more specialised dairy operations. They apply more fertiliser per hectare, which indicates a higher reliance on external inputs. They are more likely to have completed some level of agricultural education and have larger households. They also spend less on extension per cow. Among the variables presented in Table 2, we include herd size, specialisation, soil, education, age and household in the selection model to estimate propensity scores (see section 3.2 and Appendix A for more detail)^8^.

**Table 2:** Characteristics of Irish dairy farms, by technology adoption status.

Variable	Description	Non-adopters (*n* = 151)	Adopters (*n* = 145)	All farmers (*n* = 296)	Differences
*Farm characteristics*					
Herd size	Number of dairy cows	65.21 (39.41)	92.83 (40.61)	78.74 (42.26)	−5.94***
Specialisation	Ratio of dairy cows to total livestock units	0.63 (0.14)	0.66 (0.11)	0.65 (0.13)	−2.38**
Soil	= 1 if good soil quality, 0 otherwise	0.58 (0.50)	0.63 (0.49)	0.60 (0.49)	0.82 (χ^2^)
Stocking	Dairy stocking rate (number of dairy cows per hectare)	2.02 (0.55)	2.04 (0.48)	2.03 (0.52)	−0.31
Concentrates	Kg of concentrates fed per cow	916.04 (396.37)	959.25 (472.59)	937.21 (435.17)	−0.85
Fertiliser	Kg of nitrogen fertiliser applied per hectare	97.90 (50.30)	116.38 (52.57)	106.95 (52.17)	−3.09***
*Farmers’ characteristics*					
Education	= 1 if the farm holder has completed some level of agricultural education, 0 otherwise	0.69 (0.46)	0.86 (0.35)	0.77 (0.42)	12.69*** (χ^2^)
Age	Age of the farm holder	49.80 (11.32)	47.94 (10.28)	48.89 (10.84)	1.48
Household	Number of household members	3.30 (1.57)	3.72 (1.43)	3.51 (1.51)	−2.40**
Extension	Extension expenditure per cow in euro	31.64 (21.28)	27.91 (15.82)	29.82 (18.86)	1.71*

Notes: Means and standard deviations in parentheses.

Statistical tests based on *t*-tests for continuous variables and chi-square tests for binary variables (distinguished by a χ^2^).

***, **, and * significant at the 1%, 5%, and 10% level, respectively.

## 6 Results and discussion

### 6.1 Results of the treatment effect estimation

PSM results are reported in Table 3. They indicate that milk recording has a positive impact on farm economic sustainability. Firstly, it increases dairy gross margin by €54 per cow on average at the 10% significance level. Secondly, the technology increases milk yield by 406 litres per cow on average at the 1% significance level. When expressing these results in terms of percentages of potential outcome means (i.e., ${POM}^{1}$ in Table 3), we find that milk recording increases dairy gross margin and milk yield by 5% and 7% on average, respectively. When accounting for implementation costs (Irish Cattle Breeding Federation, n.d., 2020a), the estimated net benefit is €42 per cow, which translates to a 4% net increase in dairy gross margin for adopters.

**Table 3:** Estimation of Average Treatment Effects of the Treated.

Outcome variable	ATT	St. error	POM^1^	ATT as % of POM^1^
Dairy gross margin per cow (€ / cow)^a^	54.22*	32.43	1,118.13	+4.85
Milk yield per cow (l / cow)	405.57***	121.67	5,741.08	+7.06
Agricultural GHG emissions per kg of output (kg of CO_2_e / kg of FPCM)	−0.029	0.031	1.14	−2.54
Dairy nitrogen balance per ha (kg of N surplus / ha)	3.12	8.62	159.72	+1.95
BTSCC (‘000 cells / ml)	−38.86***	11.02	155.21	−25.04

**Notes**: Estimation based on propensity score matching, with two nearest neighbours.

ATT = Average Treatment Effect for the Treated; St. error = standard error; POM^1^ = potential outcome mean if all farmers were milk recording, adjusted for observables.

***, **, and * significant at the 1%, 5%, and 10% level, respectively.

a Results from the PSM procedure, prior to subtracting implementation costs.

However, the findings reveal that milk recording does not have a significant effect on farm environmental sustainability. Although we would have expected improved productivity to concurrently lead to enhanced GHG emission efficiency (Crosson et al., 2011; Guerci et al., 2013), the technology does not change GHG emitted per unit of milk produced. This suggests that the observed difference in adopters’ and non-adopters’ environmental performance (see Table 1) is not attributable to the technology’s impact, but is likely to be driven by the higher production intensity of adopters. Thus, the results show that, given current levels of data utilisation, milk recording does not directly help attenuate an environmental issue that is of increasing concern in agricultural production.

Finally, the findings in Table 3 show that milk recording is beneficial for farm social sustainability. More specifically, it has the largest effect on BTSCC, with a decrease by 38,860 cells per millilitre of milk on average at the 1% significance level. When expressing this finding as a percentage of potential outcome mean, milk recording results in a 25% reduction in BTSCC, hence improving herd health. This outcome is consistent with Dillon et al. (2016b). It is not surprising that the technology has the largest effect on herd health since its uptake is mostly promoted for the monitoring of elevated SCC (Animal Health Ireland, 2012). Indeed, farmers might be more aware and inclined to use milk recording information for herd health than for informed breeding decisions.

### 6.2 Results of the sensitivity analysis

As a robustness check, we estimate ATTs with alternative treatment-effects estimation methods and calculate the CV for each indicator. The findings are reported in Table 4. They reveal that ATT estimates do not substantially vary across IPW, RA, IPWRA and PSM estimators and that the variation around the mean remains under 16% for all indicators. It is also worth mentioning that dairy gross margin becomes significant at the 5% level with IPW and IPWRA.

**Table 4:** Sensitivity analysis to alternative treatment-effects estimation methods.

Method	ATT estimates				CV (%)
	IPW	RA	IPWRA	PSM (2NN)	
Dairy gross margin per cow (€ / cow)	64.61** (32.26)	64.93* (33.20)	63.74** (31.74)	54.22* (32.43)	8.29
Milk yield per cow (l / cow)	513.23*** (121.14)	493.71*** (115.47)	507.72*** (112.72)	405.57*** (121.67)	10.48
Agricultural GHG emissions per kg of output (kg of CO_2_e / kg of FPCM)	−0.020 (0.022)	−0.024 (0.023)	−0.028 (0.021)	−0.029 (0.031)	16.29
BTSCC (‘000 cells / ml)	−32.61** (9.44)	−27.77*** (8.52)	−32.99** (8.89)	−38.86*** (11.02)	13.73

**Notes**: ATTs and standard errors in parentheses. Coefficients of Variation (CV) calculated as a ratio of the standard deviation to the mean for the results of each indicator. IPW = Inverse-Probability Weighting; RA = Regression Adjustment; IPWRA = Inverse-Probability-Weighted Regression Adjustment; PSM = Propensity Score Matching; NN = Nearest-Neighbour. ***, **, and * significant at the 1%, 5%, and 10% level, respectively.

As PSM results may suffer from hidden bias, we estimate Rosenbaum bounds to investigate critical Γ values for dairy gross margin, milk yield and BTSCC. The findings are reported in Table 5 and show that robustness to hidden bias varies across indicators, with milk yield being the most robust, BTSCC somewhat less robust and dairy gross margin the least robust. The results suggest that our ATT estimates become sensitive to hidden bias if an unobserved characteristic causes the odds ratio of the adoption decision to differ between adopters and non-adopters by a factor of at least 2.35 for milk yield, 2.00 for BTSCC and 1.55 for dairy gross margin.

**Table 5:** Sensitivity analysis to hidden bias (Rosenbaum Bounds estimation).

Dairy gross margin per cow		Milk yield per cow		BTSCC	
Γ	(+)	Γ	(+)	Γ	(−)
1.20	0.008	1.85	0.009	1.55	0.007
1.25	0.013	1.90	0.012	1.60	0.10
1.30	0.022	1.95	0.017	1.65	0.015
1.35	0.034	2.00	0.022	1.70	0.022
1.40	0.050	2.05	0.029	1.75	0.030
1.45	0.071	2.10	0.37	1.80	0.040
1.50	0.097	2.15	0.047	1.85	0.053
1.55	0.13	2.20	0.058	1.90	0.068
		2.25	0.071	1.95	0.085
		2.30	0.085	2.00	0.11
		2.35	0.10		

**Notes**: p-values reported in the table. (+) refers to the upper bound significance levels for the overestimation of treatment effects (for indicators impacted positively by milk recording) and (-) to the lower bound significance levels for the underestimation of treatment effects (for the indicator impacted negatively by milk recording). The opposite bound significance levels were not reported as they were always above the 1% level.

We can assess whether hidden bias is a serious concern by equating the estimated critical Γ values with equivalent effects of observed characteristics from the propensity score estimation model (Model 1 in Appendix A) (DiPrete and Gangl, 2004). The three significant predictors of milk recording adoption in Model 1 are herd size, herd size squared and specialisation, with odds ratios of 1.05, 1.00 and 10.51, respectively. Concerns arise if this model specification omits important predictors that affect the adoption decision by a magnitude of at least 1.55. Given that Model 1 was not meant to perfectly predict technology adoption status and is constrained by the need to balance covariates (Caliendo and Kopeinig, 2008), it is likely to suffer from omitted variable bias if the goal is to predict the adoption decision. For this reason, we estimate a model with a wider selection of control variables^9^ and compare it to the critical Γ values. The model (reported as Model 2 in Appendix A) includes stocking rate, concentrate feed use, fertiliser usage and extension expenditure per cow in addition to the covariates from Model 1 (see Table 2 for variable description). The results show that the odds ratios of the additional significant variables (stocking rate and fertiliser use) remain under 1.01, thus revealing that their exclusion from the propensity score estimation does not challenge our PSM results.

Concerns can also arise from the exclusion of unobservables such as farmers’ motivation and ability, but we control for these characteristics through education level, extension expenditure and degree of dairy specialisation. This is based on the idea that better-informed, more-commercially oriented farmers are likely to be more motivated and inclined to adopt new technologies (Feder et al., 1985; Sauer and Zilberman, 2012). Effectively, it is unlikely that an unobserved confounder would be a stronger predictor of the adoption decision than the variables included in Model 2. Therefore, while it cannot be ruled out that selection occurs also on unobservables, the study provides evidence of an impact of milk recording on economic and social farm sustainability.

## 7 Conclusions and policy implications

In recent years, the sustainability of agricultural production has moved to the forefront of public concerns and the political agenda. While this is a topical issue for many agricultural sectors worldwide, lessons can be learned from the Irish dairy sector, which is currently undergoing rapid growth initiated by EU milk quota abolition. This article evaluates the ‘win-win-win’ potential of an agricultural technology, i.e., milk recording, to simultaneously enhance all dimensions of farm sustainability and thereby foster sustainable intensification on Irish dairy farms. We apply matching methods to a representative sample of 296 farms to control for observed farm and farmers’ characteristics that affect the adoption decision and estimate treatment effects on a wide set of sustainability indicators.

Our empirical findings show that milk recording enhances farm economic and social sustainability through a 4% net increase in dairy gross margin, a 7% improvement in milk yield and a 25% reduction in BTSCC. The technology’s impact on BTSCC suggests a decrease in the risk of mastitis incidence due to the relationship between elevated SCC and mastitis (Geary et al., 2012; Huijps et al., 2010b; Sharma et al., 2011). Therefore, this study supports the idea that technology adoption can reconcile productivity and animal welfare objectives at the farm level (Dawkins, 2017).

Conversely, we did not find a significant impact of milk recording on farm environmental sustainability, as measured by GHG emission efficiency of milk production. This result suggests that productivity gains reached through milk recording may not be sufficient to dilute the GHG costs of animal maintenance (Crosson et al., 2011; Guerci et al., 2013). Alternatively, these productivity gains might have been achieved through enhanced reliance on external inputs (Foote et al., 2015), which can counteract GHG benefits of improved efficiency (Basset-Mens et al., 2009). Increases in productivity per additional unit of external inputs need to be larger so that GHG emission efficiency is overall improved (Crosson et al., 2011). Consequently, considering the current application of milk recording, this study does not confirm the technology’s ‘win-win-win’ potential to foster a sustainable intensification of Irish milk production.

Important policy implications arise from this study. The results suggest that increasing milk recording’s adoption rates would be valuable to increase output and enhance animal health for farmers who are not currently milk recording. The technology implies an out-of-pocket expenditure, whose return on investment can be difficult to assess for farmers. While milk recording adoption does not imply a direct cash return, our findings confirm clear economic benefits. In that sense, the study offers interesting insights in terms of methodological approach by extending the application of farm sustainability indicators for the measuring of technology impact. The estimation of treatment effects on sustainability indicators is a means of isolating the impact of farm strategies and provides evidence-based, self-explanatory figures (e.g., 25% decrease in BTSCC) that can subsequently inform farmers’ adoption decisions. The diffusion of our research findings to farmers through veterinarians and extension agents would be useful to encourage milk recording’s uptake. These actors are important sources of knowledge for farmers (Genius et al., 2014; Sligo and Massey, 2007; Vrain and Lovett, 2016) and are already actively involved in the promotion of ‘best’ practices in Ireland (see for instance Animal Health Ireland, n.d., 2020a, b; Department of Agriculture Food and the Marine, n.d, 2020).

While milk recording is a support technology, farmers remain the central piece in the system as they are the ones making decisions and acting upon the delivered information (Berckmans, 2014; Hostiou et al., 2017). Our data does not allow us to assess the extent or manner in which milk recording information is utilised to inform farm management decisions and further research is needed investigate this topic. Even though milk recording organisations provide training and support to implement the technology and interpret results, concerns may arise from the large amount of information returned to farmers (Progressive Genetics, n.d., 2020) since it might difficult to select which of it is key for decision making (Hostiou et al., 2017; Schewe and Stuart, 2015).

Previous research (e.g., Dillon et al., 2018) has indicated that farmers adopt a short-term reactionary (versus precautionary) approach towards mastitis management and as such milk recording technology might not be widely used for breeding decisions. Using the information for this purpose might potentially require a much deeper understanding of the figures and expected impacts on cow offspring. If it were improved, herd productivity could be further enhanced^10^. Increases in GHG emission efficiency could also be expected with significant improvements in herd genetic merit (Lanigan et al., 2018), if excessively high levels of external inputs are avoided (Crosson et al., 2011). This accentuates the role of individual decision making based on milk recording information and need of further training, notably through extension. More emphasis on all potential applications of milk recording information (including breeding) may improve current depth of use by milk recorders as there seems to be scope for improvement. ‘Information intensive technologies’ (i.e., which provide information to support decision making) (Barnes et al., 2019) tend to require further investments in training and learning so that farmers use them at full potential (Barnes et al., 2019; Eastwood et al., 2012).

In absence of proven GHG benefits, it might be difficult to justify public intervention to support the promotion of milk recording for environmental purposes alone (Barnes et al., 2019). Nevertheless, it is still likely to gain policy interest in the short-term future in the frame of the new EU regulation addressing the public risk of antimicrobial resistance (European Parliament Council of the European Union, 2019; Irish Co-operative Organisation Society, 2019). This regulation will come into force in January 2022. One of its goals is to reduce preventative antibiotic use in livestock production. As a result, strict restrictions on the use of ‘blanket dry cow therapies’ will be implemented at dry-off so that only dairy cows for which it is an absolute necessity to use dry cow antibiotics will receive the treatment (Irish Co-operative Organisation Society, 2019). Farmers will have to move towards ‘selective dry cow therapies’ and distinguish cows that qualify for dry cow strategies free from antibiotics (i.e., internal teat sealants) (AHI, n.d. b). Milk recording is one tool that can help farmers identify cows with low risks of infection at dry-off (through individual SCC readings) and thus comply with the new regulation (Animal Health Ireland, n.d., 2020a, b; Irish Co-operative Organisation Society, 2019). Thus, the technology might contribute even more to the social sustainability of Irish milk production by helping to prevent antimicrobial resistance. Up until January 2022, adoption rates must increase (Irish Co-operative Organisation Society, 2019), thus justifying public intervention, for instance, through subsidised trials. If farmers are to fully bear the costs of this new regulation, more research is needed to understand barriers to the adoption of milk recording. In the meantime, the figures estimated in this study can be used to encourage voluntary uptake.

Finally, sustainable intensification is likely to rely on more controlled agricultural systems, with minimal waste along the supply chain. As dairy farmers upgrade their milking equipment, there will be opportunities to encourage a move away from traditional types of support technologies like milk recording, on to more sophisticated precision livestock farming technologies like automated milking facilities (Eastwood et al., 2012). These can provide daily information for herd monitoring and thus real-time decision aid, with expectations of economic, environmental and social sustainability benefits (Barnes et al., 2019; Berckmans, 2014; Eastwood et al., 2012). Just as for milk recording, the realisation of their ‘win-win-win’ potential to achieve sustainable intensification will depend on the actual use of the information they provide to inform daily decision making (Berckmans, 2014; Eastwood et al., 2012; Hostiou et al., 2017). Therefore, more research is needed to improve the link between technology adoption and farm management decisions.

## Declaration of Competing Interest

None.

## Figures and Tables

**Table A1:** Estimation of adoption decision models (logit regressions).

Covariate	Odds ratio	
	Model 1	Model 2
Herd size	1.05*** (0.011)	1.06*** (0.012)
Herd size squared	1.00*** (0.000047)	1.00*** (0.000047)
Specialisation	10.51** (11.06)	7.42* (8.12)
Soil	1.02 (0.27)	1.08 (0.30)
Education	1.16 (1.86)	1.07 (1.78)
Age	1.00 (0.025)	1.00 (0.026)
Education * Age	1.01 (0.030)	1.01 (0.031)
Household	1.10 (0.098)	1.11 (0.10)
Stocking		0.48** (0.15)
Concentrates		1.00 (0.00032)
Fertiliser		1.01** (0.0030)
Extension		1.00 (0.0083)
Constant	0.0059*** (0.010)	0.0092** (0.018)
Pseudo R^2^	0.15	0.17
Log-likelihood	−174.83	−170.41
Observations	296	296
Overlap region	[0.064; 0.85]	N.A.

**Notes**: Results reported as odds ratios and standard errors in parentheses. Model 1 is the propensity score estimation model and Model 2 is used for comparison purposes in section 5.2. N.A. = Non-Applicable. ***, **, and * significant at the 1%, 5%, and 10% level, respectively.

**Fig. B1:**
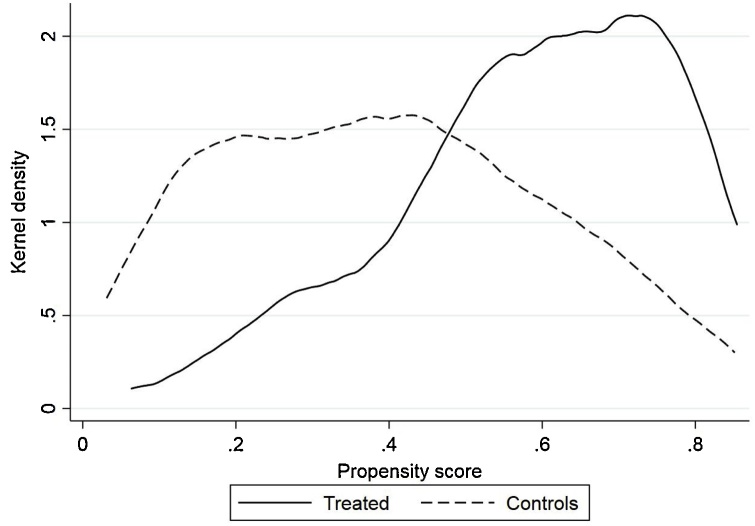
Kernel density distribution of propensity score, by treatment status.

**Table C1:** Standardised differences between both groups before and after propensity score matching (in %).

	Original	Matched
Herd size	69.00	−0.94
Herd size squared	48.71	−2.07
Specialisation	27.74	−2.55
Soil	10.48	−6.46
Education	42.32	−6.18
Age	−17.24	10.01
Education * Age	35.44	0.44
Household	27.96	−2.75
Total reduction in bias	34.86	3.93
Number of observations	296	290
Treated observations	145	145
Control observations	151	145

**Fig. D1:**
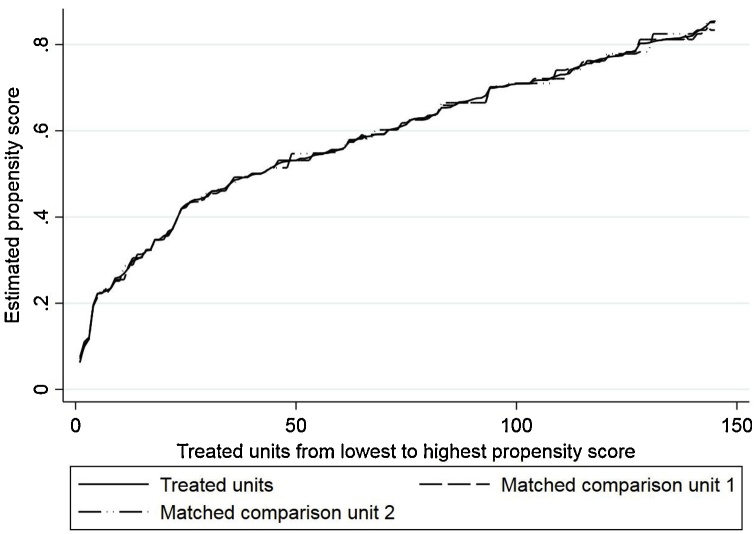
Propensity scores for treated and matched comparison units after matching, lowest to highest.

## Appendix A Adoption decision models

See Table A1

## Appendix B Overlap assumption

See Fig. B1

## Appendix C Balancing property

See Table C1

## Appendix D Matching quality

Fig. D1

## Footnotes

1 In 2010, the period of ‘soft landing’ started and milk quotas were increased by 1% every year until 2015.

2 Non-ETS sectors include agriculture, transport, residential, commercial, waste and non-energy intensive industry.

3 Age has a post-matching standardised difference of 10.01%. However, it is kept in the model as it improves the balance across other covariates that play a greater role in the adoption decision model (Lee, 2013).

4 Other numbers of matches investigated during the exploration process included one to four non-adopters. Moreover, matching without replacement was not a viable option in this analysis as the reservoir of non-adopters relative to adopters is small (151 vs. 145 observations).

5 More specifically, the NFS fulfils Ireland’s statutory obligation as a EU Member State to provide the European Commission with financial and technical data relating to agriculture.

6 The LCA methodology used in this study was implemented according to the publicly-available PAS 2050:2011 specification from the British Standards Institute (BSI, 2011) and validated by the Carbon Trust, an accredited third party (O’Brien et al., 2014a).

7 For a full description of the GHG emission sources and corresponding emission factors, see O’Brien et al. (2014a).

8 Some variables from Table 2 are not included in the propensity score model specification as their inclusion does not lead to improved covariate balance. Nevertheless, we estimate another adoption decision model including all variables to assess whether hidden bias is of concern in section 5.2. It is reported as Model 2 in Appendix A.

9 The model specification is based on previous literature focusing on technology adoption (Feder et al., 1985; Läpple et al., 2017; Sauer and Zilberman, 2012).

10 Age at first calving is between 24 and 36 months in Ireland (Berry and Cromie, 2009), thus pointing out the time lag between current breeding decisions and resulting herd performance improvements.
